# Development of a Deep Learning Model to Automatically Identify Palatal Landmarks on Three‐Dimensional Maxillary Dental Casts

**DOI:** 10.1155/ijod/9409391

**Published:** 2026-02-12

**Authors:** Jamal Giri, George Vadakepurathan Jose, Nikhil Cherian Kurian, Alan Brook, Lyle Palmer, Toby Hughes

**Affiliations:** ^1^ Adelaide Dental School, The University of Adelaide, Adelaide, South Australia, Australia, adelaide.edu.au; ^2^ Australian Institute for Machine Learning, The University of Adelaide, Adelaide, South Australia, Australia, adelaide.edu.au; ^3^ School of Public Health, The University of Adelaide, Adelaide, South Australia, Australia, adelaide.edu.au

## Abstract

**Objectives:**

To develop a deep learning model to automatically identify palatal landmarks on three‐dimensional (3D) digital maxillary dental casts, and to evaluate model performance.

**Materials and Methods:**

Twelve palatal landmarks were manually annotated on each 3D digital maxillary dental cast from 377 individuals in the permanent dentition stage. Manually annotated landmarks were used as ground truth to develop and to evaluate a deep learning model for automatic landmark detection. A two‐stage PointNet++ architecture was employed, where coarse landmark predictions were first generated, followed by localized refinement for improved precision. The model accuracy was evaluated by measuring the linear discrepancy between the final predicted and the ground‐truth landmark positions.

**Results:**

A PointNet++‐based hierarchical deep learning model, designed to extract both local and global features from point clouds, was developed. The model demonstrated a mean landmark detection error of 0.55 mm (SD ± 0.49) between predicted and ground‐truth positions across 12 landmarks. The model also exhibited high predictive performance, correctly predicting 90% of landmarks within 1 mm and 98% within 2 mm of the ground truth.

**Conclusions:**

A deep learning model was developed for automated identification of 12 palatal landmarks on 3D maxillary dental casts, which demonstrated high performance. Our model will enable more efficient morphological assessment of the palate by substantially reducing the time for manual annotation in clinical and research settings.

## 1. Introduction

The palate is an important structure in the craniofacial complex. Palatal morphology has drawn considerable clinical attention, as it varies across different types of malocclusions and is frequently altered during orthodontic intervention [1]. Palatal morphology is routinely evaluated during orthodontic assessments using dental casts, primarily through palatal depth and width measurements.

With the advent of three‐dimensional (3D) digital dental casts and sophisticated software, the scope of morphological analysis has greatly expanded [2]. High‐resolution 3D digital casts enable a better evaluation of the palate with greater accuracy, which is not limited to linear measurements and includes the measurements of surface area and volume of the palate [3]. Semi‐landmarks, points that slide along curves or surfaces to capture shape, can be automatically placed on digital casts to evaluate the palatal shape, using manually annotated anatomical landmarks as a reference [4]. While digitization has made the analysis more efficient, the process still relies on the manual identification of anatomical landmarks by an expert. Manual landmarking using handcrafted features remains the gold standard [5]. However, manual landmarking is time‐consuming and prone to fatigue‐induced errors [6]. Automating landmark identification on dental casts could improve measurement accuracy and efficiency, benefiting both clinicians and researchers.

In recent years, deep learning models have been used for automated landmark identification in medical imaging, including orthodontic applications [7, 8]. Several studies have explored automated applications for identifying landmarks on lateral cephalometric radiographs and CBCT images; however, research on their application in identifying palatal landmarks on 3D dental casts is limited [8]. While a geometric deep learning model showed promising accuracy in automatically identifying seven palatal landmarks on 3D maxillary casts, its performance was notably less reliable for mid‐palatal points [9]. The need to visually validate landmarks and make corrections limits the suitability of such models for fully automated applications [9]. Using only seven palatal landmarks does not capture the full complexity of palatal morphology. The absence of benchmark datasets and evaluation frameworks for palatal landmarking on 3D dental casts further hinders reproducibility and cross‐study comparisons, highlighting the need for a robust deep learning model capable of generalizing across diverse anatomical variations.

We hypothesized that replacing less reliable mid‐palatal landmarks with more identifiable points along the gingival margins of the teeth, corresponding to specific teeth from central incisor to first molar on both sides, totaling 12 landmarks, would improve deep learning model accuracy in capturing palatal morphology. Therefore, the objectives of the current study were to train a deep learning model to automatically identify 12 palatal landmarks on 3D digital maxillary casts and to test model accuracy in identifying these landmarks.

## 2. Materials and Methods

### 2.1. Samples

A total of 377 3D digital maxillary dental casts in the permanent dentition stage were obtained from the archives of the Craniofacial Biology Research Laboratory at Adelaide Dental School [10]. The samples were individuals of European descent with no craniofacial anomalies. Dental casts of the participants with no history of orthodontic treatment or restorations, such as crowns, were included in the study. The 3D digital dental casts were generated by scanning the stone dental casts using a 3D laboratory scanner (E4 3Shape, Copenhagen, Denmark) in Standard Tessellation Language (.STL) format, with an accuracy of 4 μm. For model development, the digital casts were randomly divided into training (251 casts), validation (63 casts), and test (63 casts) sets. Ethics approval was obtained from the Human Research Ethics Committee at the University of Adelaide (Approval Number: H‐2023‐060).

### 2.2. Landmark Annotation

**Figure 1:**
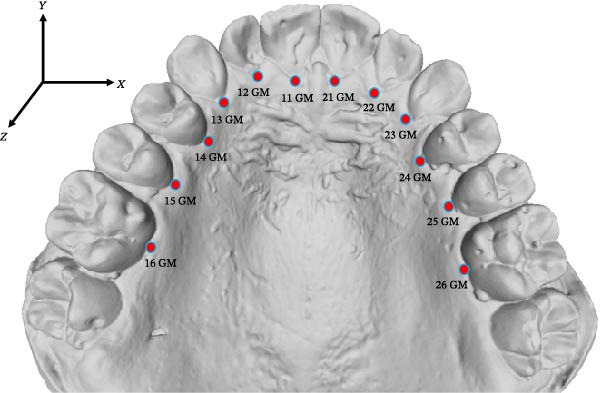
A maxillary dental cast with palatal landmarks.

Twelve palatal landmarks were annotated on each 3D digital maxillary dental cast in MeshLab (ISTI‐CNR, Pisa, Italy, version 2022.02) using the PickPoints tool (Figure 1 and Table 1). The landmarks, manually annotated by a single examiner (JG) with 11 years of clinical experience, were used as the ground truth for model training, validation and testing. Forty randomly selected 3D dental casts were re‐annotated by the same examiner after 2 weeks to evaluate the intraexaminer reliability of ground truth.

**Table 1:** List of palatal landmarks.

Landmark (GM)	Description
16	The most apical point on the palatal gingival margin of the right first molar (16)
15	The most apical point on the palatal gingival margin of the right second premolar (15)
14	The most apical point on the palatal gingival margin of the right first premolar (14)
13	The most apical point on the palatal gingival margin of the right canine (13)
12	The most apical point on the palatal gingival margin of the right lateral incisor (12)
11	The most apical point on the palatal gingival margin of the right central incisor (11)
21	The most apical point on the palatal gingival margin of the left central incisor (21)
22	The most apical point on the palatal gingival margin of the left lateral incisor (22)
23	The most apical point on the palatal gingival margin of the left canine (23)
24	The most apical point on the palatal gingival margin of the left first premolar (24)
25	The most apical point on the palatal gingival margin of the left second premolar (25)
26	The most apical point on the palatal gingival margin of the left first molar (26)

Abbreviation: GM, gingival margin.

### 2.3. Pre‐Processing

Each 3D digital maxillary dental cast represented a surface mesh of the maxillary permanent dentition and palate, containing between 60,000 and 120,000 vertices. To prepare the dataset for analysis, each point cloud was first aligned by rotating it to ensure uniform orientation and then scaled to fit within a normalized bounding box of [−1, 1] along its longest axis. Farthest Point Sampling was applied to uniformly downsample the point cloud to 24,000 points, preserving a representative and evenly distributed subset of points while enhancing computational efficiency [11].

### 2.4. Model Architecture

The model employed a two‐stage framework for accurate landmark localization, consisting of a coarse prediction stage followed by a refinement stage.

#### 2.4.1. Coarse Prediction Stage

This stage used a PointNet++[12]‐based encoder to extract hierarchical geometric features from an input point cloud of size [24,000 × 6], containing vertex positions and normals. The encoder comprised three set abstraction layers with increasing query radii (0.025, 0.1, and 0.2), enabling multiscale feature extraction. Encoded features were passed through two parallel 1D convolutional heads. One predicted point‐to‐landmark distances, while the other estimated directional offsets from input points to target landmarks. These outputs were combined to generate an initial set of 256 candidate landmark points. Training of the coarse prediction stage was guided by a composite loss function comprising distance loss, chamfer loss, and separation loss (Table S1).

#### 2.4.2. Refinement Stage

The refinement stage improved prediction accuracy by focusing on localized regions around each landmark. The 256 coarse points were grouped into 12 clusters using density‐based spatial clustering of applications with noise (DBSCAN), each corresponding to a potential landmark. A 6 mm geodesic ball was then extracted around each cluster centroid to form a localized patch containing structural detail, as geodesic distances better capture the palate’s curved anatomy than straight‐line measures.

These patches were processed using a PointNet++ encoder–decoder network. The encoder reduces complex 3D data into essential information, while the decoder reconstructs the original shape using that information. The encoder included three set abstraction layers for feature extraction, while the decoder used three feature propagation layers to upsample features back to the original resolution. A probability map was generated for each patch, and the final landmark was identified as the point with the highest predicted probability.

### 2.5. Training and Testing

The model was trained using PyTorch [13], an open‐source machine learning framework, with an NVIDIA RTX 2080 Ti GPU. Training of the coarse prediction module was conducted over 50 epochs using the Adam optimizer with a learning rate set to 0.001. The refinement module was also trained for 50 epochs but used a higher learning rate of 0.002 and gradient clipping, with a threshold of 0.5 to stabilize training. The model was tuned based on its validation set performance. Final evaluation was conducted on a held‐out test set, to assess generalization to unseen data.

### 2.6. Model Accuracy

Landmark localization absolute error was calculated by comparing the model’s predicted (*x*, *y*, *z*) coordinates against ground‐truth manual annotations. The Euclidean distance between each predicted landmark and its corresponding ground truth was calculated to determine the distance error. The model’s overall accuracy was then quantified by computing the mean error distance across all landmarks. The success detection rate (SDR) was calculated to determine the percentage of predicted landmarks within specified distance thresholds from the ground truth for each landmark. These thresholds were set at ≤ 0.5, 1, 1.5, and 2 mm.

## 3. Results

The mean intraexaminer error in manual landmark identification across all 12 landmarks was 0.34 ± 0.06 mm (range: 0.25–0.5 mm) (Table S2a). The ICC for landmark coordinates was greater than 0.93 (range: 0.93–0.99), suggesting high reproducibility (Table S2b).

**Figure 2:**
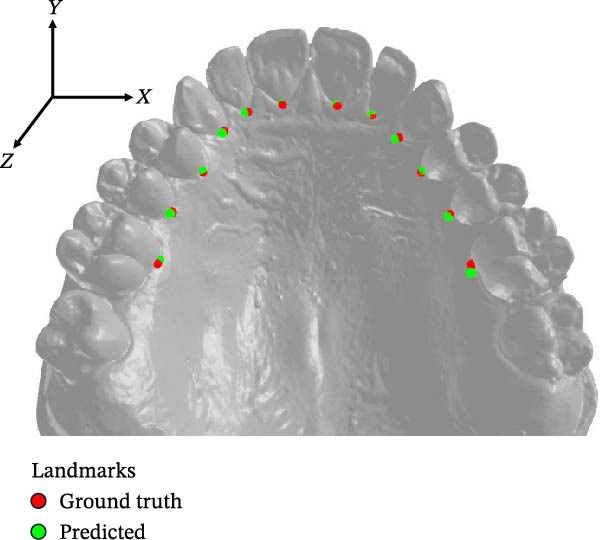
Predicted and ground truth landmarks on a maxillary dental cast.

**Figure 3:**
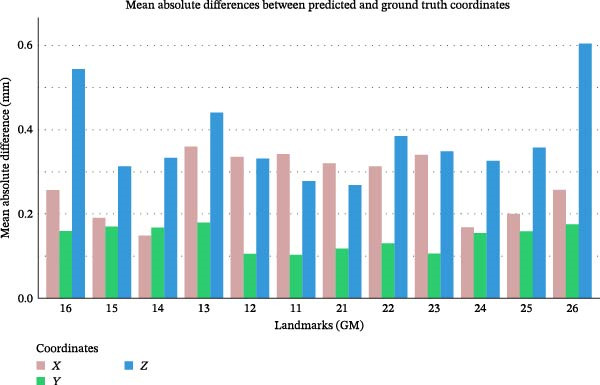
Landmark localization error along each axis (*x*, *y*, *z*) for all landmarks.

Figure 2 displays predicted and ground‐truth landmark positions of the 12 landmarks on a maxillary cast. The mean absolute error for all 12 landmarks was highest along the *z*‐axis (0.37 ± 0.39 mm) while it was lowest along the *y*‐axis (0.14 ± 0.16 mm) (Figure 3).

**Figure 4:**
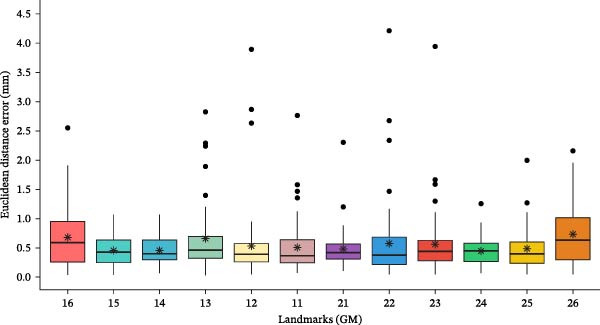
Box plot of landmark localization error for each landmark, with mean Euclidean error indicated by stars and outliers represented by dots.

The mean Euclidean distance error between the predicted landmarks and their corresponding ground‐truth positions across all 12 landmarks was 0.55 ± 0.49 mm (Table 2). Landmarks related to premolar teeth (14, 15, 24, and 25 GM) showed low mean localization errors, ranging from 0.45 to 0.48 mm. In contrast, landmarks associated with the first molars exhibited larger mean localization errors, with 0.73 mm for 26 GM and 0.67 mm for 16 GM. While the mean localization error for each landmark remained below 1 mm, some outliers displayed localization errors as high as 4.5 mm for certain landmarks (Figure 4).

**Table 2:** Mean Euclidean distance and success detection rates within 2 mm for predicted landmarks compared to ground truth.

Landmarks (GM)	Euclidean distance (mm)		Success detection rate % (SDR %)			
	Mean	SD	0.5 mm	1 mm	1.5 mm	2 mm
16	0.67	0.51	47.62	76.19	93.65	98.41
15	0.46	0.25	60.32	96.83	100.00	100.00
14	0.46	0.24	63.49	98.41	100.00	100.00
13	0.66	0.76	55.56	87.30	92.06	93.65
12	0.53	0.63	63.49	95.24	95.24	95.24
11	0.50	0.44	63.49	90.48	96.83	98.41
21	0.48	0.32	61.90	96.83	98.41	98.41
22	0.57	0.66	58.73	90.48	95.24	95.24
23	0.56	0.55	58.73	88.89	95.24	98.41
24	0.45	0.23	63.49	98.41	100.00	100.00
25	0.48	0.36	61.90	88.89	98.41	100.00
26	0.73	0.51	39.68	73.02	90.48	98.41
Overall	0.55	0.49	58.20	90.08	96.30	98.02

Abbreviation: GM, gingival margin

Overall, 90% of predicted landmarks were within 1 mm of their ground truth positions, and 98% were within 2 mm. For landmarks 14, 15, and 24 GM, the SDR within 1.5 mm was 100%, while for 25 GM, 100% of predicted landmarks were within 2 mm of their ground truth positions (Table 2).

## 4. Discussion

We have developed a PointNet++‐based deep learning model for the automated identification of 12 palatal landmarks on 3D dental casts. Our model demonstrated high accuracy, with a mean landmark detection error of 0.55 mm and a 90% SDR within 1 mm, highlighting its feasibility for accurate landmark localization for both clinical and research purposes.

The accuracy of our model was better than an earlier model [9], which automatically identified seven palatal landmarks with a median landmarking error between 0.78 and 1.45 mm. The earlier study [9] found that midpalatal landmarks were particularly problematic, with even expert annotators struggling to identify them accurately, resulting in large error rates. Landmarks that are more prone to errors in manual identification also display larger errors in automated detection [14]. Thus, we excluded midpalatal landmarks from our model and increased the number of dentogingival landmarks, which are easier to annotate, to 12 to enable a more comprehensive evaluation of the palate. Similarly, palatal rugae were not selected as reference landmarks due to their questionable stability and susceptibility to change during orthodontic procedures such as palatal expansion [15]. The midpalatal landmarks can be mathematically computed from accurately identified bilateral dentogingival landmarks using a robust singular value decomposition method [16] that accounts for the asymmetry present in the dental arches.

The accuracy of automated landmark identification on 3D dental casts varies depending on the type of landmark. The average localization errors for occlusal landmarks, such as cusp tips and incisal edges, range between 0.3 and 2.4 mm [17–21]. Greater localization errors have been reported for cervical landmarks as compared to occlusal landmarks [22]. On 2D lateral cephalograms, a detection rate exceeding 85% within 2 mm is considered clinically acceptable, while an error of less than 1 mm is considered accurate [23, 24]. An error threshold of less than 2 mm is widely accepted as clinically acceptable in dentistry [25]. Given these thresholds, our model, with a mean landmark detection error of 0.55 mm and a 90% detection rate within 1 mm, demonstrates clinically acceptable accuracy in landmark localization, ensuring reliable palatal analysis. However, even a 2 mm discrepancy can have significant clinical implications for some specific landmarks, emphasizing the need to maintain the highest possible accuracy [26]. Training the model with larger and more diverse datasets will further improve its accuracy, enhancing its reliability for research and clinical applications.

The accuracy of our deep learning model can be largely attributed to the hierarchical learning approach of PointNet++[12], which improves its feature extraction process. Unlike traditional PointNet [27], which captures only global features, PointNet++ employs a multiscale hierarchy that samples and groups points into local regions. This enables the model to extract detailed geometric features at different resolutions and combine them into a comprehensive representation. This allows the model to identify fine features like gingival margins within the broader palatal shape. This hierarchical learning improves the model’s ability to handle noise and variability in palatal morphology, enabling sufficiently accurate landmark identification for clinical applications.

The quality of the ground‐truth data significantly influences the model’s performance in landmark detection [28]. High‐quality ground truth annotations are crucial for training models that can accurately localize landmarks on dental casts. High ICC (> 0.93) and low mean Euclidean distance error (0.34 mm) for all landmarks suggest a strong intraexaminer reproducibility for our ground truth annotations. A common assumption in machine learning is that training and test data come from the same distribution; however, in the real world, this assumption often does not hold [29]. This discrepancy can adversely affect model performance. In our test set, certain dental casts exhibited large errors for specific landmarks, presenting as outliers in the boxplots (Figure 4). These discrepancies likely arise from differences between the training and test datasets. Natural variations in gingival margin morphology, along with inflammation or recession of the marginal gingiva, may have compromised the model’s ability to accurately identify those landmarks. Notably, outliers with large errors were associated with labially erupted canines and their adjacent lateral incisors. Although large errors were infrequent, such outliers may have clinical implications, potentially leading to inaccurate assessment of palatal morphology and influencing treatment planning. Incorporating additional training data with these characteristics could enhance the model’s accuracy. The large error rate for landmarks associated with molars may be due to poorly defined palatal gingival concavities resulting from their greater mesiodistal width.

Our model utilizes PointNet++ to directly analyze the 3D point cloud, unlike some landmark identification methods that convert 3D dental casts into 2D projections and use heatmaps for processing with conventional convolutional neural networks [19, 30]. While 2D methods reduce computational complexity, they risk losing crucial depth and spatial information, which can in turn compromise accuracy, especially for landmarks in curved or occluded regions of dental casts. By preserving the full 3D geometry, our hierarchical learning model captures both local details and global context, enhancing the model’s accuracy.

Our deep learning model shows promising accuracy for identifying palatal landmarks; however, training on larger datasets may further improve its accuracy [31]. Unfortunately, orthodontic research often operates in institutional silos, with limited data sharing and few public repositories of 3D dental casts [32]. Data sharing is often linked to privacy concerns, but it is possible to share data while maintaining privacy [33]. Collaborative data sharing and standardized benchmarks are essential for developing more robust machine learning models for both clinical and research applications.

To simplify the model development, maxillary dental casts with all permanent teeth up to the first permanent molars were used. Our model could underperform in dental arches with multiple missing teeth, severe crowding, and ectopically erupted teeth. In the future, we intend to develop a robust model that can automatically identify palatal landmarks in primary, mixed, or permanent dentition stages with high accuracy.

While the current study highlights the potential of a deep learning model for automated palatal landmark detection, it also has some limitations. These include the model’s use of dental casts from European ancestry with permanent dentition, without craniofacial anomalies, such as cleft palate, and using a single annotator. Another limitation of this study is the reliance on tooth‐dependent landmarks derived from a complete dentition, which may restrict the model’s applicability in patients with missing and extracted teeth. While the midpalatal landmarks, which are tooth independent, were excluded due to high estimation error, future research should focus on improving midline landmark estimation and identifying a reduced subset of highly repeatable landmarks that are applicable across a wider range of dentitions. Future work should (1) expand datasets to include dental casts representing diverse ancestries and clinical conditions; (2) incorporate annotations from multiple clinicians to establish robust consensus standards; and (3) validate the model across different 3D scanning systems. Additionally, optimizing the model for real‐time processing could further enhance its clinical adoption.

## 5. Conclusions

This study developed a hierarchical deep learning model based on the PointNet++ architecture to automatically identify palatal landmarks on 3D dental casts. The model enables efficient assessment of palatal morphology, substantially reducing manual effort and observer variability. This automation enables the analysis of large datasets to advance research on palatal growth and morphology, while also improving the efficiency of diagnosis and treatment planning in clinical orthodontics. While the current model focuses on a defined set of palatal landmarks, future work should extend its application to a wider range of anatomical regions and more diverse clinical datasets to enhance its generalizability and clinical utility.

## Funding

This study was supported by the National Health and Medical Research Council (Australia) (Grant APP157904), the Australian Dental Research Foundation (Grant ADRF 08/2012), and the University of Adelaide Paul Kwok Lee Bequest (Grants PKL 75131608, PKL 75131612, and PKL 75134783). Open access publishing facilitated by Adelaide University, as part of the Wiley ‐ Adelaide University agreement via the Council of Australasian University Librarians.

## Disclosure

The funding support did not influence the study design, data collection, analysis, interpretation, or publication decisions.

## Ethics Statement

The study was approved by the Human Research Ethics Committee at the University of Adelaide (Approval Number: H‐2023‐060).

## Conflicts of Interest

The authors declare no conflicts of interest.

## Supporting Information

Additional supporting information can be found online in the Supporting Information section.

## Supporting information


**Supporting Information** Table S1. Description of loss functions. Table S2a. Euclidean distances between landmarks based on repeated manual annotations. Table S2b. Intraexaminer reliability for repeated manual annotations.

## Data Availability

The data that support the findings of this study are available from the corresponding author upon reasonable request.
