# Supplementary material for: Development of a Deep Learning Model to Automatically Identify Palatal Landmarks on Three‐Dimensional Maxillary Dental Casts
Source: Int J Dent. 2026 Feb 12;2026:9409391. doi: 10.1155/ijod/9409391 (PMC12900578; doi:10.1155/ijod/9409391)
Supplement: Supplementary file 1 — Supporting Information Table S1. Description of loss functions. Table S2a. Euclidean distances between landmarks based on repeated manual annotations. Table S2b. Intraexaminer reliability for repeated manual annotations. [file IJOD-2026-9409391-s001.doc]

Table 1: Description of loss functions

| **Loss function** | **Description** |
| --- | --- |
| Distance loss | Minimizes the Euclidean distance between each predicted point and its nearest ground truth landmark, ensuring spatial proximity to the true landmark locations. |
| Chamfer loss | Refines predictions by enforcing a bijective correspondence between predicted points and landmarks. Ensures every landmark is represented by at least one prediction (surjection), and each prediction maps uniquely to one landmark (injection), eliminating redundancy. |
| Separation loss | Enhances prediction distinctiveness by maximizing the ratio of the distance to the closest landmark over the distance to the second closest. This discourages ambiguous associations and promotes tight clustering around correct landmarks. |

Table 2a: Euclidean distances between landmarks based on repeated manual annotations

| **Landmarks** | **Euclidean Distance (mm)** | |
| --- | --- | --- |
|  | **Mean** | **Standard deviation** |
| 16 GM | 0.50 | 0.55 |
| 15 GM | 0.32 | 0.22 |
| 14 GM | 0.36 | 0.19 |
| 13 GM | 0.34 | 0.24 |
| 12 GM | 0.37 | 0.33 |
| 11 GM | 0.37 | 0.29 |
| 21 GM | 0.31 | 0.18 |
| 22 GM | 0.33 | 0.18 |
| 23 GM | 0.30 | 0.19 |
| 24 GM | 0.25 | 0.23 |
| 25 GM | 0.32 | 0.19 |
| 26 GM | 0.38 | 0.42 |

Table 2b: Intra-examiner reliability for repeated manual annotations

| **Landmarks** | **Coordinates** | **ICC** |
| --- | --- | --- |
| 16 GM | x | 0.981 |
| 16 GM | y | 0.985 |
| 16 GM | z | 0.940 |
| 15 GM | x | 0.994 |
| 15 GM | y | 0.990 |
| 15 GM | z | 0.980 |
| 14 GM | x | 0.994 |
| 14 GM | y | 0.988 |
| 14 GM | z | 0.971 |
| 13 GM | x | 0.988 |
| 13 GM | y | 0.994 |
| 13 GM | z | 0.973 |
| 12 GM | x | 0.992 |
| 12 GM | y | 0.997 |
| 12 GM | z | 0.930 |
| 11 GM | x | 0.990 |
| 11 GM | y | 0.996 |
| 11 GM | z | 0.950 |
| 21 GM | x | 0.993 |
| 21 GM | y | 0.997 |
| 21 GM | z | 0.980 |
| 22 GM | x | 0.989 |
| 22 GM | y | 0.998 |
| 22 GM | z | 0.985 |
| 23 GM | x | 0.994 |
| 23 GM | y | 0.998 |
| 23 GM | z | 0.988 |
| 24 GM | x | 0.995 |
| 24 GM | y | 0.995 |
| 24 GM | z | 0.989 |
| 25 GM | x | 0.995 |
| 25 GM | y | 0.984 |
| 25 GM | z | 0.993 |
| 26 GM | x | 0.995 |
| 26 GM | y | 0.990 |
| 26 GM | z | 0.976 |

ICC: Intra-class correlation coefficient
